# Safety for older adults using telecare: Perceptions of homecare professionals

**DOI:** 10.1002/nop2.328

**Published:** 2019-07-01

**Authors:** Torunn Beate Johannessen, Marianne Storm, Anne Lise Holm

**Affiliations:** ^1^ Faculty of Health and Social Sciences Western Norway University of Applied Sciences Haugesund Norway; ^2^ Department of Public Health, Faculty of Health Sciences University of Stavanger Stavanger Norway

**Keywords:** homecare professionals, homecare services, older adults, safety, telecare

## Abstract

**Aim:**

The aim of this study was to explore homecare professionals' perceptions of safety related to the use of telecare by older adults.

**Design:**

An exploratory qualitative design was employed.

**Methods:**

Two focus group interviews with ten female homecare professionals (nine Registered Nurses and one occupational therapist) were carried out between June–December 2017. The participants were recruited from six community homecare services in two Norwegian municipalities. Data were analysed using qualitative content analysis.

**Results:**

The participants perceived that the use of telecare protects older adults against injury and insecurity by preventing harm and giving them a feeling of safety. However, they also stated that the use of telecare involves challenges that could lead to harm to older adults due to technological limitations and difficulties managing and understanding the technology. Although telecare can enhance safety, it is necessary to develop reliable technology and adapt it to the user's abilities, skills and resources.

## 1 INTRODUCTION

Use of telecare technology has the potential to maintain and enhance older adults' independence and quality of life, reduce hospital and care home admissions and enable them to remain in their own homes for a longer time (Botsis, Demiris, Pedersen, & Hartvigsen, 2008; Milligan, Roberts, & Mort, 2011). Telecare has also been identified as an important tool for addressing predicted future challenges caused by the larger proportion of older people in the population and the worldwide workforce shortage (European Commission, 2010). Telecare is the use of information, communication and monitoring technologies that allow healthcare professionals to remotely evaluate health status, provide educational interventions or deliver health and social care to patients in their homes (Solli, Bjørk, Hvalvik, & Hellesø, 2012: p. 2802). By enabling healthcare professionals to provide care at a distance to patients' homes, telecare represents a significant shift in the way that care services are provided (Oudshoorn, 2012).

During the last decade, several developed countries have begun to implement telecare through different local and national initiatives (Milligan et al., 2011). In Norway, an overarching national strategy is to integrate telecare in the community health and care services by 2020 (Ministry of Health & Care Services, 2013). As a result, a national programme for the development and implementation of telecare was established to facilitate co‐operation and exchange of experiences between municipalities that use and integrate telecare as a part of the community health and care service. Since the start of the programme in 2013, several Norwegian municipalities have participated in various implementation projects covering a range of home‐based telecare technologies such as localization technology (GPS), electronic medicine dispensers, digital camera supervision and web‐based portals for people with various chronic diseases (Directorate of Health, 2019). In most cases, the telecare device is managed by the patients themselves or with the help of their family carers. However, homecare professionals in the community homecare service follow‐up telecare use among older adults as well as the alerts received from, for example mobile safety alarms, fall alarms and electronic medicine dispensers. Homecare professionals also have remote conversations with older adults using web portals, in addition to supervising them remotely by means of digital cameras. Hence, homecare professionals must be considered an important source of empirical knowledge about telecare use among older adults.

More advanced age is associated with a greater risk of injury and harm. According to the World Health Organization (WHO), the major burdens of disability and death arise from age‐related losses of hearing, sight and movement by the age of 60 years. Higher age also implies an increased risk of many health disorders such as chronic respiratory conditions and dementia (WHO, 2015). People aged over 60 years are also at greater risk of falls (WHO, 2018) and medication errors (Barber et al., 2009; Fialová & Onder, 2009). Hence, ensuring the safety of patients is a major area of concern for those delivering healthcare worldwide (WHO, 2017), where the use of technology in healthcare is considered to have a positive impact on patient safety by reducing the risk of human error (Ball, Weaver, & Abbott, 2003). Ensuring the safety of older adults who live at home is also a highly important aim in the effort to implement telecare in community health and care services in Norway (NOU, 2011; Ministry of Health & Care Services, 2013). Patient safety is defined as the avoidance, prevention and amelioration of adverse outcomes or injuries stemming from the process of health care (Vincent, 2010: p. 32). Most previous research on patient safety has been conducted in a hospital setting and not in a primary healthcare context where most care is delivered (WHO, 2017). The use of telecare in homecare is an expanding research area (Lindberg, Nilsson, Zotterman, Siv Söderberg, & Skär, 2013). There is also an extensive global interest in exploiting the potential of digital technologies to enhance the quality and safety of health care (Black et al., 2011). However, a previous review of health technologies and their impact on the quality and safety of healthcare delivery identified a large gap between the postulated and empirically demonstrated benefits (Black et al., 2011). Furthermore, another review performed to identify patient safety risks associated with telecare use in homecare identified a need for more research to avoid or minimize potential harm to patients (Guise, Anderson, & Wiig, 2014). Qualitative research concerning safety in telecare use has addressed specific telecare interventions such as safety alarms (Melander‐Wikman, Fältholmand, & Gard, 2008; Melkas, 2010), fall detectors and bed occupancy sensors (Horton, 2008) and smoke detectors (Doughty & Orton, 2014). Other studies have been performed to describe safety experiences of telecare use from the perspectives of persons with dementia and their family carers (Riikonen, Mäkelä, & Perälä, 2010; Olsson, Engström, Skovdahl, & Lampic,^2012^ 2012). Studies have also found that home healthcare nurses who delivered health services through virtual visits evaluated the virtual visit technology positively (Husebø & Storm, 2014). Moreover, in a study performed by Barrett (2017) aimed at understanding how teleconsultation has an impact on the role of nurses, it was reported that nurses have different types of presence (operational, clinical, technical and social) during teleconsultation to support patient care. The degree of presence depends on specific characteristics of video‐mediated communication. However, few qualitative studies have addressed the safety of older adults who use telecare from the perspective of homecare professionals. More knowledge can lead to increased focus on aspects that may be of importance for the safety of older adults who use telecare at home.

### 1.1 Aim

The aim of this study was to explore homecare professionals' perceptions of safety related to the use of telecare by older adults. The research question was as follows: How do homecare professionals perceive safety in relation to older adults' use of telecare?

## 2 METHOD

### 2.1 Study design

An explorative qualitative research design (Polit & Beck, 2012) was used to obtain the participants' perceptions. A qualitative design is concerned with producing discursive descriptions and exploring social actors' meanings and interpretations (Blaikie, 2010: p. 204). Data were collected by means of two focus groups, which is a qualitative research technique that enables the collection of data through group interaction on a topic determined by the researcher (Morgan, 1996).

### 2.2 Sample

The recruitment aimed to ensure a sample with the greatest amount of insight to illuminate the presented topic (Krueger & Casey, 2015). To be included in the study, the participants had to have a minimum of six months' work experience with telecare devices used by older adults who receive community care in their own homes. Homecare professionals who met the inclusion criteria were asked to participate by a municipal contact person (one department manager and one Telecare department employee) in each of the municipalities.

The sample comprised of ten female homecare professionals recruited from two Norwegian municipalities (one large and one medium‐sized). Both municipalities were participating in the national programme for the development and implementation of telecare (Directorate of Health, 2019) and over the previous few years had implemented a range of different home‐based telecare devices for older adults living at home. The participants in focus group A consisted of four Registered Nurses (RNs) and one occupational therapist (OT) recruited from five different homecare services using different telecare devices. The participants in focus group B comprised of five Registered Nurses (RNs) recruited from one homecare service. The participants had between 6–32 months of work experience with ten different telecare devices, presently or previously in use by older adults in the two municipalities. The telecare devices are presented in Table 1.

**Table 1:** Overview and description of the telecare devices

Focus group/municipality	Telecare device	Description
A	Web portal	Web‐based portal for remote communication between homecare professionals and patients with chronic obstructive pulmonary disease (COPD). Also possibilities for video conversations
A	Ambient control technology	Ambient control technology for doors, light, heat, door phone, windows, curtains and sun shielding in patients' homes
A + B	Mobile safety alarm	GSM‐based, wearable alarm for outdoor use with emergency button, loudspeaker and Global Positioning System (GPS) for localizing patients
A + B	Electronic medicine dispenser	Electronic medicine dispenser with medication reminder. If the medication is not released from the dispenser in a given time, the dispenser automatically locks and alerts homecare professionals
A + B	Digital camera	Digital camera for remote supervision of patients living at home. Mostly in use at night. Takes “snapshots” at an arranged time
A + B	Light sensor	Motion sensor for switching lights on/off
A + B	Door exit sensor	Automatically alerts homecare professionals if patients open the door. Available with a voice messenger
B	Bed sensor	Automatically alerts homecare professionals if the patient does not return to bed in a pre‐determined time
B	Smoke detector	Automatically alerts homecare professionals if smoke develops in the home
B	Fall sensor	Wearable. Automatically alerts homecare professionals if patients fall at home. The patient can also press an emergency button

### 2.3 Data collection

The focus groups interviews were carried out between June–December 2017. The interviews took place in meeting rooms in the municipality where the participants had their respective workplaces. The first author (TBJ) served as moderator in both focus group interviews, while the co‐authors (MS and ALH) each acted as co‐moderator in one group, observing the dynamics and social interaction between group members (Morgan, 1996). Both focus group interviews were based on a semi‐structured interview guide including the following request; *“*Can you please discuss how the use of telecare ensures safety for older adults?” The group discussion comprised both descriptions of the telecare devices and a sharing of perceptions and experiences of the topic. All the participants contributed to the discussions and were encouraged by the moderators to freely share their perceptions. The moderators added in‐depth supplementary open‐ended questions when necessary. Each focus group interview lasted approximately 1–1.5 hr. Both interviews were audio recorded and transcribed verbatim shortly after they had been conducted.

### 2.4 Analysis

A qualitative content analysis as described by Graneheim and Lundman (2004) was conducted. Qualitative content analysis is described as a systematic approach for classifying and identifying themes or patterns in the data (Hsieh & Shannon, 2005). All three authors participated in the entire analysis process. In the first step, the authors carefully read through the transcribed material several times to gain a sense of the whole. In the second step, the authors searched for meaning units in the text. In the third step, the identified meaning units were condensed while preserving their main content. In the fourth step, the condensed meaning units were labelled with a code. In the fifth step, the codes were sorted into categories based on their similarities and differences. Finally, the categories were abstracted into two themes on a descriptive level (Graneheim, Lindgren, & Lundman, 2017). The three authors discussed the themes and categories until consensus was achieved.

### 2.5 Ethical considerations

The study was conducted in accordance with the Declaration of Helsinki (WMA, 2013). The study was pre‐approved by the Norwegian Social Science Data Service (project number 48429). Prior of the focus group interviews, the participants were provided with both written and oral information about the study and written informed consent was obtained. The participants' confidentiality was ensured by anonymization and the confidential handling of the data. A more detailed description of the participants' workplace is omitted to avoid identification.

## 3 RESULTS

The analysis resulted in two descriptive themes. An overview of categories and sub‐categories is presented in Table 2. In the following, the results will be presented with selected representative quotations to illuminate the participants' perceptions and the analysis process.

**Table 2:** Themes and categories

Themes	Categories
A protection against injury and insecurity	Preventing harm
	Feeling safe
Involves challenges that could lead to harm	Technological limitations
	Difficulties managing and understanding the technology

### 3.1 A protection against injury and insecurity

This theme illuminates that the participants believed that telecare can protect older adults against injury and insecurity. The theme is based on two categories: *Preventing harm* and *Feeling safe*.

#### 3.1.1 Preventing harm

The participants perceived that telecare prevents harm to older adults. For example, they related that they had several call‐outs due to the occurrence of smoke at service users' homes that are equipped with smoke detectors. One participant stated:

We see that it has averted situations. We've had a real proper fire and it was the smoke detector that first alerted us. (Nurse 6)

Furthermore, the participants mentioned that the GPS in the mobile safety alarm had been used successfully several times to localize missing persons with dementia. According to one participant, in other municipalities older adults with dementia died outdoors because they were unable to find their way home. A participant revealed that before the use of mobile safety alarms, homecare personnel often had to search outdoors for patients. A participant said:

Although they do not the press the emergency button we can see where they are if we do not find them at home in the evening. (Nurse 7)

The participants stated that the main purpose of the web portal is preventing the exacerbation of COPD, thus avoiding hospital admissions. According to the participants, the use of a video conversation provides an important opportunity for clinical observation of COPD status. As one of the participants expressed:

You observe a lot when you are talking to them face to face, even if it is through the net. (Nurse 2)

It was also agreed that using electronic medicine dispensers prevents harm to older adults. However, it was emphasized that in most cases medicine dispensers are not suitable for persons with dementia. According to the participants, the dispenser ensures the correct dose at the right time by reminding the patient when to take the medication and remotely alerting homecare professionals if the medication is not released from the dispenser in a given time. The medicine dispenser was also perceived to prevent patients from taking too much medication as it automatically locks if the dose is not released. A participant stated:

It is very safe that way. There are few medication errors with that dispenser. (Nurse 1)

#### 3.1.2 Feeling safe

The participants found that telecare promotes an increased feeling of safety among older adults. The mobile safety alarm was especially highlighted as increasing the sense of safety for persons with dementia. As one participant commented:

They feel safe when they have GPS and can find their way home. Because there have been episodes when they did not return home after going out. (Nurse 7)

The participants also revealed that they often receive feedback that the service users feel safer with the safety alarm as they can receive help outdoors when necessary. As one of the participants related:

A couple we visit were very happy to be picked up because they had gone further than they could really cope with health wise and could not find their way home again. (Nurse 7)

Another participant revealed:

A woman I care for is very happy that she can use the mobile safety alarm because it makes her feel more secure. She does not think it is so nice to go outside alone, but at the same time she wants to have the freedom to go whenever she wants. (Nurse 4)

The participants also perceived that service users with COPD felt safer having contact with homecare professionals through the web portal. One stated:

I think that patients feel a lot safer by signing up on the web portal. And even if the line breaks, we'll call them on the phone (Nurse 3)

Another explained:

I have the impression that it makes them feel safe and that they like it when we phone and have time to chat with them because they can tell us how they feel and talk a little. (Nurse 2)

### 3.2 Involves challenges that could lead to harm

This theme demonstrates that the participants were of the opinion that telecare involves challenges that could lead to harm to older adults. The theme is based on two categories: *Technological limitations* and *Managing and understanding the technology*.

#### 3.2.1 Technological limitations

The participants perceived that limitations in the technology could lead to harm to older adults. For example, a participant reported that she had experienced that one of the digital cameras placed in a patient's home did not work for a whole weekend due to poor mobile network coverage in the area. Another participant related:

We have a camera that goes on and off all the time, even though the plugs are in and all. (Nurse 5)

Furthermore, the participants reported that they often experienced an unstable Internet connection when holding video conversations with service users suffering from COPD, where the net often breaks up, stops and vibrates. According to the participants, this stresses the service users. As one of the participants stated:

They become stressed. They are overjoyed to get through and be in touch and then the connection breaks down and you have to do it all over again. It is bad for the COPD patients we are working for. (Nurse 2)

The participants emphasized that when Internet problems occur, homecare professionals always phone the service users instead. However, a participant underlined that a telephone conversation does not provide an opportunity for clinical observation:

We do not get that: ‘Yes, you make an effort when you breathe,’ ‘What is the skin colour like?’ If they are in poor shape you do not get the visual impression of how they are (Nurse 3)

The participants also revealed that even though GPS generally picks up the exact location of lost and missing adults, the GPS positioning disappears if the patient is in an area covered by trees, in a building or in a car. As a participant explained:

When they are driving a car example, they are locked in and the GPS signals will not be picked up. And if they go into a building or are in a place with lots of trees their position disappears. So, there are some limitations with the usability of that device. (Nurse 4)

Another perceived safety challenge was related to the use of fall sensors. A participant elaborated that the alarm sometimes does not trigger if the older adult collapses without a sudden movement, while on the other hand it can easily go off due to a strong movement. Moreover, if the patient remains lying on the floor or begins to move after falling, the alarm stops beeping. According to the participants, a consequence of these issues is that some service users often stop using the alarm and put it away. A participant described the problem as follows:

It's so sensitive that it often goes off and they become annoyed and put it away because it is so easy to activate. Then they are not safe. (Nurse 8)

However, the participants emphasized that the development of personal fall sensor technology is complex due to the many different ways of falling. Hence, not many fall sensors have been employed in recent years.

#### 3.2.2 Difficulties managing and understanding the technology

The participants found that difficulties managing and understanding the technology could lead to harm to older adults. According to the participants, many persons diagnosed with dementia have great difficulty relating to, managing and understanding the functions of the mobile safety alarm. As one participant explained:

The patients with dementia are not always able to handle the functions of the mobile safety alarm. They do not manage to push the emergency button and speak into it themselves or understand its functions. (Nurse 9)

The participants also reported that when they dial the patients on the alarm and it automatically connects, some of those suffering from dementia do not understand where the voice is coming from. A participant described:

On one occasion there was a lady who had wandered off. She eventually ended up at the Emergency Department where they pressed the emergency button and got in touch with us. So that user group is always a challenge. (Nurse 4)

Another perceived safety challenge was related to the use of ambient controlling technology of, for example doors, lights and curtains. According to the participant, while healthy service users had no problem mastering the technology, those with dementia found it more difficult to manage and understand:

It didn't go very well because this technology is supposed to be used more actively and it may not work smoothly when it is hard to learn new things. (Occupational therapist)

In addition, persons with dementia found sensors difficult to manage and understand. The participants revealed that the pre‐recorded voice messenger on the door exit sensor often made some of them confused and anxious. One participant described it as follows:

Yes, that voice sensor when you go out of the door: ‘Now it is night. Go to bed,’ If the voice comes from a stranger, they often become anxious. If it is a familiar voice, they also become anxious: ‘Huh, wasn't it my daughter?’ And every time you go in or out of the door, there's someone talking to you, so we took it away. (Nurse 9)

The light sensors were also found to cause difficulties for persons with dementia. As a participant explained:

Some didn't understand what happened and why the light went on. Most patients are used to turning the light on and off and when it was the opposite, they found it difficult to relate to. Many patients spent a long time sitting still on the toilet and then suddenly it went completely dark. (Nurse 4)

The participants also mentioned that some of the service users turned off the door exit sensor themselves because they did not want to bother the homecare personnel. Additionally, they experienced that some disconnect their telecare devices due to their habit of unplugging all electrical devices in the evening. The participants revealed that if several telecare devices are connected, this will disconnect not only the sensors but also the alarms. Moreover, it occasionally occurs that the service users turn off the alarms themselves. A participant stated:

We had a lady who had a direct connection to the Fire Department, but if the fire alarm went off, she just took a broom and beat the alarm off. (Nurse 9)

## 4 DISCUSSION

This study aimed to explore homecare professionals' perceptions of safety related to the use of telecare by older adults. The first theme reveals that the participants perceived that the use of telecare protects older adults against injury and insecurity. In particular, the use of mobile safety alarms and video conversations promoted a feeling of security among service users. Electronic medicine dispensers, mobile safety alarms and the web portal were also highlighted as technologies that prevented harm and injury to older adults. These findings are supported by several previous studies. For example, a study performed by Melander‐Wikman et al. (2008) found that an increased feeling of safety was a significant reason for using mobile safety alarms among older adults. Furthermore, a study conducted by Melkas (2010) noted that safety alarms have a positive impact on perceived health due to improved safety. Other studies have shown that the use of various types of telecare technology can have a positive impact on the safety of people with dementia and their family carers (Gibson, Dickinson, Brittain, & Robinson, 2015; Olsson et al., 2012; Riikonen et al., 2010). Safety barriers can be explained as “physical or non‐physical means planned to prevent, control or mitigate undesired events or accidents” (Sklet, 2006:496). According to Reason (1997, 2000), people create different barriers (defences) to prevent accidents from occurring, which can be either “soft” (e.g., procedures and training) or “hard” (e.g., technical devices and alarms). Reason (1997) demonstrates that the purpose of these barriers is to stand between the hazard and the potential losses (e.g., people), thus preventing an adverse event, or reducing its consequences. The concept of safety barriers can be applied to illuminate how telecare can be understood as a physical barrier to prevent or reduce the consequences of adverse events that may cause harm to older adults. Consequently, the use of telecare can reduce older adults' need for hospital admission, residential care or other public care services and enable them to live for a longer time in their own homes. Additionally, it may empower them to undertake more physical and social activities outside of the home, thus enhancing their quality of life. Hence, an possible implication for the homecare services is that telecare can be a significant tool for enhancing patient safety and addressing the safety needs of older adults.

The second theme reveals that the participants perceived that the use of telecare involves challenges that could lead to harm to older adults. The participants perceived limitations in the technology related to the use of mobile safety alarms, the web portal, digital cameras and fall alarms. Perceived difficulties in managing and understanding the technology were especially associated with the fact that many older adults either did not understand or were incapable of managing the functions of the mobile safety alarm, the ambient controlling technology and the sensor devices. A central finding was that managing and understanding the technology was especially problematic for older adults with dementia. These findings are in line with several previous studies. For example, a review performed by Bharucha et al. (2009) on the use of technology in dementia care found that much still remains to be done to design technologies that are functional and acceptable for users with dementia. Furthermore, a previous study demonstrated that people with dementia accepted telecare devices more readily if they were easy to use (Riikonen et al., 2010). Studies have also shown that telecare technology can play an important role in health care if the devices are adapted to users' needs (Hoonakker, Khunlertkit, Tattersall, Keevil, & Smith, 2012). Unreliable technology and difficulty understanding its functions may lead to harm to older adults in several ways. For instance, inability to understand the functions of a mobile safety alarm may result in the user failing to call for help in the case of an emergency. Studies also report that limitations associated with the technology, such as technical failure, can inhibit the uptake and adoption of the technology by nurses (Barrett, 2017). Moreover, if older adults repeatedly find the technology unreliable or difficult to understand, they may develop a negative attitude towards the telecare device and not bother using it. We should also bear in mind that inadequate technology may lead to a false sense of security, not only for the users but also for their relatives and homecare personnel. It is therefore vital that the technology can meet the users' safety expectations. According to Sklet (2006), a successful barrier function should have a direct and significant effect on the occurrence and/or consequences of an adverse event or accident. However, Reason (1997) shows that an adverse event can occur due to weaknesses in the defences caused by active failures (e.g., unsafe acts by personnel) and latent conditions (e.g., poor design, inadequate tools). This perspective can be used to illuminate the vulnerability of telecare because safety is dependent on the technology working properly, being used correctly and suitably designed for the users. Hence, a potential implication for ensuring the safety of older adults who use telecare might be to promote and facilitate the development of robust and reliable information and communications technology (ICT) systems and telecare technology. Furthermore, it may also be of importance to bear in mind that although the devices themselves might appear to have simple functions, some older adults may nevertheless experience challenges and difficulties using them. It is therefore necessary that telecare use by older adults is closely followed up by the homecare services and that the telecare solutions offered are adapted to each user's individual abilities, skills and resources.

### 4.1 Study limitations

This study has several potential limitations. Firstly, the focus group participants had relevant work experience with the ten telecare devices that had been implemented in the two municipalities. However, it is likely that participants who only had experience of a smaller number of telecare devices would have led to a deeper discussion about each of the devices. Another possible limitation is related to the composition of the focus groups. In the group that consisted of participants who did not know each other, some participants may have been reluctant to share all their thoughts and perceptions, while in the other group where the participants were colleagues, it is possible that they expressed more consensus than would have been the case with strangers. Moreover, although telecare is a new research area for the authors, it is possible that our pre‐understanding (Gadamer, 2004) as healthcare researchers and Registered Nurses may have influenced the analysis process.

## 5 CONCLUSION

The participants perceived that the use of telecare protects older adults against injury and insecurity by preventing harm and giving them a feeling of safety. However, they also stated that the use of telecare involves challenges that could lead to harm to older adults due to technological limitations and difficulties managing and understanding the technology. The study indicates that telecare can be a significant tool for enhancing patient safety and addressing the safety needs of older adults. To ensure the safety of older adults who use telecare, the study underlines the need for the development of robust and reliable information and communications technology (ICT) systems and telecare technology. Furthermore, it is necessary that telecare use by older adults is closely followed up by the homecare services and that the telecare solutions offered are adapted to each user's individual abilities, skills and resources.

## CONFLICTS OF INTEREST

The authors declare that there is no conflict of interest in this study.

## AUTHOR CONTRIBUTIONS

The study design was developed by TBJ and ALH. All authors contributed to the data collection and analysis. The manuscript was drafted by TBJ, while ALH and MS contributed to the preparation and revision of the manuscript. All authors read and approved the final version of the manuscript.
